# Assessment of Neonatal Abstinence Syndrome Surveillance — Pennsylvania, 2019

**DOI:** 10.15585/mmwr.mm7002a3

**Published:** 2021-01-15

**Authors:** Kathleen H. Krause, Joann F. Gruber, Elizabeth C. Ailes, Kayla N. Anderson, Victoria L. Fields, Kimberlea Hauser, Callie L. Howells, Allison Longenberger, Nancy McClung, Lisa P. Oakley, Jennita Reefhuis, Margaret A. Honein, Sharon M. Watkins

**Affiliations:** ^1^Epidemic Intelligence Service, CDC; ^2^National Center on Birth Defects and Developmental Disabilities, CDC; ^3^Pennsylvania Department of Health; ^4^National Center for Immunization and Respiratory Diseases, CDC; ^5^Department of Research & Evaluation, Kaiser Permanente Southern California, Pasadena, California.

The incidence of neonatal abstinence syndrome (NAS), a withdrawal syndrome associated with prenatal opioid or other substance exposure (*1*), has increased as part of the U.S. opioid crisis (*2*). No national NAS surveillance system exists (*3*), and data about the accuracy of state-based surveillance are limited (*4*,*5*). In February 2018, the Pennsylvania Department of Health began surveillance for opioid-related NAS in birthing facilities and pediatric hospitals* (*6*). In March 2019, CDC helped the Pennsylvania Department of Health assess the accuracy of this reporting system at five Pennsylvania hospitals. Medical records of 445 infants who possibly had NAS were abstracted; these infants had either been reported by hospital providers as having NAS or assigned an *International Classification of Diseases, Tenth Revision, Clinical Modification* (ICD-10-CM) hospital discharge code potentially related to NAS.^†^ Among these 445 infants, 241 were confirmed as having NAS. Pennsylvania’s NAS surveillance identified 191 (sensitivity = 79%) of the confirmed cases. The proportion of infants with confirmed NAS who were assigned the ICD-10-CM code for neonatal withdrawal symptoms from maternal use of drugs of addiction (P96.1) was similar among infants reported to surveillance (71%) and those who were not (78%; p = 0.30)*.* Infants with confirmed NAS who were not assigned code P96.1 typically had less severe signs and symptoms. Accurate NAS surveillance, which is necessary to monitor changes and regional differences in incidence and assist with planning for needed services, includes and is strengthened by a combination of diagnosis code assessment and focused medical record review.

Five Pennsylvania hospitals were selected to represent various sizes, geographic regions, and anticipated NAS incidence. A broad NAS case definition was used to identify infants who possibly had NAS under the a priori assumption that hospitals might not always assign an infant P96.1 or a clinical diagnosis of NAS, despite the presence of NAS symptoms. Infants who possibly had NAS were aged <28 days born during March 1–August 31, 2018, and either reported to NAS surveillance or assigned a hospital discharge ICD-10-CM code indicative of prenatal substance exposure or NAS symptom.^§^ Medical records of all infants who possibly had NAS were reviewed for demographic and birth characteristics, prenatal opioid and other substance exposure, infant and maternal toxicology results and NAS symptoms and treatment information. Infants were considered to have confirmed NAS if all of the following criteria were documented in the infant medical record: 1) at least one NAS symptom; 2) maternal history or toxicology results indicating prenatal opioid exposure; and 3) a clinical mention of NAS (i.e., NAS listed in the discharge diagnosis or problem list or use of a NAS scoring tool [e.g., Finnegan]). For infants with confirmed NAS, maternal prenatal and delivery records were abstracted to gather additional data on prenatal opioid or other substance exposure.

Sensitivity and positive predictive value (PPV) of the Pennsylvania NAS surveillance system were calculated, with corresponding 95% confidence intervals (CIs) estimated using an exact binomial distribution. Descriptive analyses compared infants with confirmed NAS by reporting status and by presence of ICD-10-CM code P96.1. Categorical variables were compared using chi-squared tests (or Fisher’s exact tests for cell counts <5); continuous variables were compared using negative binomial regression. Statistical significance was assessed at *a *= 0.05. All analyses were conducted using SAS (version 9.4; SAS Institute). This activity was reviewed by CDC and was conducted consistent with applicable federal law and CDC policy.^¶^

Overall, 445 infants who possibly had NAS were identified: 192 were reported to surveillance and 253 identified through diagnosis codes alone (Figure). Medical record review identified 241 infants with confirmed NAS, 191 of whom were reported to surveillance (sensitivity = 79% [191 of 241; 95% CI = 74%–84%]; PPV = 99% [191 of 192; 95% CI = 97%–100%]). Among the 241 infants with confirmed NAS, those reported to surveillance were significantly more likely than were those not reported to have documentation of neonatal (69% versus 50%) or maternal (55% versus 30%) toxicology evidence of prenatal opioid exposure in the infant record, maternal history of prenatal opioid exposure in the maternal record (98% versus 90%), and prenatal exposure to cannabis (30% versus 10%) in the infant or maternal record (Table 1). Notably, 71% of infants reported to surveillance were assigned ICD-10-CM code P96.1, which was not significantly different from infants not reported (78%).

**FIGURE:**
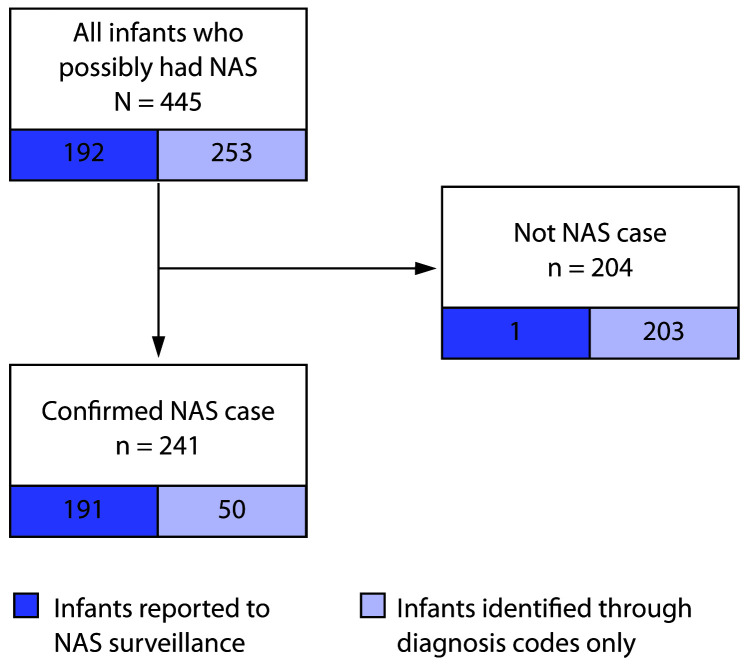
Identification of infants with confirmed neonatal abstinence syndrome (NAS) through medical record review of those reported to NAS surveillance and those identified by diagnosis codes — selected hospitals, Pennsylvania, 2018

**TABLE 1:** Characteristics of infants with confirmed neonatal abstinence syndrome (NAS) based on medical record review (N = 241) who were reported and not reported to surveillance — selected hospitals, Pennsylvania, 2018

Characteristic	No.* (%) or mean (range)			p-value^†^
	All infants with NAS (N = 241)	Infants reported to surveillance (N = 191)	Infants not reported to surveillance, identified through diagnosis codes only (N = 50)	
**Maternal race**				
White	211 (91)	171 (92)	40 (83)	0.055
Other^§^	22 (9)	14 (8)	8 (17)	
**Maternal ethnicity**				
Hispanic or Latina	2 (>1)	1 (1)	1 (2)	0.361
Not Hispanic or Latina	223 (99)	179 (99)	44 (98)	
**Source of payment in maternal record**				
Medicaid	216 (93)	174 (94)	42 (91)	0.530
Private/Other	16 (7)	12 (6)	4 (9)	
**Maternal age, yrs**	234^¶^; 29 (18–43)	184^¶^; 29 (18–43)	50^¶^; 30 (22–40)	0.112
**Infant sex**				
Male	118 (49)	97 (51)	21 (42)	0.269
Female	123 (51)	94 (49)	29 (58)	
**Gestational age, wks**	235^¶^; 38 (32–42)	187^¶^; 38 (32–41)	48^¶^; 37 (32–42)	0.417
**Type of hospitalization**				
Birth hospitalization	221 (92)	178 (93)	43 (88)	0.208
Other type of admission	19 (8)	13 (7)	6 (12)	
**Length of stay, days**	240^¶^; 13 (1–68)	190^¶^; 13 (2–68)	50^¶^; 12 (1–47)	0.596
**NAS scores**				
Age at first NAS score, days	234^¶^; 1 (0–19)	186^¶^; 1 (0–17)	48^¶^; 2 (0–19)	0.163
First NAS score**	239^¶^; 3 (0–19)	190^¶^; 3 (0–14)	49^¶^; 4 (0–19)	0.063
Age at highest NAS score, days	230^¶^; 5 (0–32)	182^¶^; 5 (0–32)	48^¶^; 4 (1–21)	0.275
Highest NAS score**	238^¶^; 10 (2–21)	189^¶^; 10 (2–21)	49^¶^; 10 (2–19)	0.659
**Symptoms**				
Total number of symptoms^††^	240^¶^; 11 (1–17)	191^¶^; 12 (1–17)	49^¶^; 11 (1–17)	0.147
**Evidence of prenatal opioid exposure in the infant record** ^§§^				
Neonatal toxicology evidence	157 (65)	132 (69)	25 (50)	0.012
Maternal toxicology evidence	120 (50)	105 (55)	15 (30)	0.002
Maternal history	225 (93)	178 (93)	47 (94)	1.000
**Evidence of prenatal opioid exposure in the maternal prenatal or delivery record^§§^**				
Maternal toxicology evidence	56 (23)	44 (23)	12 (24)	0.886
Maternal history	233 (97)	188 (98)	45 (90)	0.011
**Type of opioid exposure^¶¶^**				
Buprenorphine	160 (66)	125 (65)	35 (70)	0.544
Methadone	68 (28)	58 (30)	10 (20)	0.147
Opiates, unspecified	69 (29)	57 (30)	12 (24)	0.416
Heroin	40 (17)	35 (18)	5 (10)	0.159
Oxycodone	30 (12)	22 (12)	8 (16)	0.393
Other opioids***	17 (7)	12 (6)	5 (10)	0.361
**Type of other exposure** ^†††^				
Tobacco	179 (74)	146 (76)	33 (66)	0.133
Cannabis	63 (26)	58 (30)	5 (10)	0.004
Cocaine	38 (16)	34 (18)	4 (8)	0.126
Antidepressants	35 (15)	25 (13)	10 (20)	0.217
Benzodiazepines	34 (14)	24 (13)	10 (20)	0.179
Amphetamine	27 (11)	23 (12)	4 (8)	0.614
Gabapentin	22 (9)	17 (9)	5 (10)	0.810
**Infant receipt of pharmacologic treatment for NAS**				
Yes	107 (44)	87 (46)	20 (42)	0.567
No	129 (54)	101 (54)	28 (58)	
**ICD-10-CM discharge diagnosis codes^§§§^**				
P96.1, Neonatal withdrawal symptoms from maternal use of drugs of addiction	174 (72)	135 (71)	39 (78)	0.304
P04.1, Newborn (suspected to be) affected by other maternal medication (2018 edition code)	8 (3)	5 (3)	3 (6)	0.368
P04.2, Newborn affected by maternal use of tobacco	10 (4)	7 (4)	3 (6)	0.437
P04.3, Newborn affected by maternal use of alcohol	1 (0)	0 (—)	1 (2)	0.207
P04.41, Newborn affected by maternal use of cocaine	5 (2)	4 (2)	1 (2)	1.000
P04.49, Newborn (suspected to be) affected by maternal use of other drugs of addiction	80 (33)	64 (34)	16 (32)	0.840

**Abbreviations:** ICD-10-CM = *International Classification of Diseases, Tenth Revision, Clinical Modification*.

* Frequencies might not sum to total because of missing values.

^†^ P-values were calculated comparing infants reported to surveillance with infants not reported to surveillance using a negative binomial likelihood ratio test for continuous variables and chi-squared test (or Fisher’s exact test, if at least one cell count was <5) for categorical variables.

^§^ Other races included were Black, Asian, Native Hawaiian/Other Pacific Islander, American Indian/Alaska Native, not specified, or unknown. Given the small denominator in each category, all were collapsed into a single category.

^¶^ Data are not available for all members of this cohort.

** Includes all infants with a recorded score. All scores were Finnegan or modified Finnegan.

^††^ Signs and symptoms include tremors, breathing problems, blotchy skin, diarrhea, crying, fever, fussiness, gagging or retching, hiccups, hyperactive or exaggerated Moro reflex, frequent yawning, overactive reflexes, poor feeding, salivation, seizures, skin abrasions or excoriation, slow weight gain, sneezing, stuffy nose, suckling issues, sweating, vomiting, increased muscle tone, trouble sleeping, and any other symptom attributed to NAS by a clinician.

^§§ ^Not mutually exclusive categories.

^¶¶ ^As documented in the maternal record, infant record, or both.

*** Other opioids include codeine, fentanyl, hydrocodone, hydromorphone, kratom, morphine, and tramadol.

^††† ^As documented in the maternal record, infant record, or both. Other substances with <20 infants exposed included alcohol, antipsychotics, barbiturates, bupropion, methamphetamine, phencyclidine, and other substances referred to directly, such as “methaqualone,” or indirectly, such as “maternal polysubstance abuse.”

^§§§ ^No infants were assigned ICD-10-CM code P96.2, “Withdrawal symptoms from therapeutic use of drugs in newborn,” or P04.40, “Newborn affected by maternal anesthesia and analgesia in pregnancy, labor and delivery.”

Among infants with confirmed NAS, type and source of opioid exposure were similar in those who were and were not assigned P96.1 (Table 2). However, infants assigned P96.1 were more likely than were those not assigned P96.1 to have mothers enrolled in Medicaid (95% versus 88%), significantly longer lengths of stay (14 versus 9 days), older ages at first NAS score (2 versus 1 days), higher first NAS scores (4 versus 2), older ages at peak NAS score (5 versus 3 days), higher peak NAS scores (11 versus 9), more NAS symptoms (12 versus 9), more frequent pharmacologic treatment (61% versus 3%), and greater prenatal exposure to gabapentin in the infant or maternal record (12% versus 1%). Infants not assigned P96.1 were significantly more likely to be assigned ICD-10-CM code P04.49, “Newborn suspected to be affected by maternal use of other drugs of addiction” (60% versus 23%).

**TABLE 2:** Characteristics of infants with confirmed neonatal abstinence syndrome (NAS) based on medical record review (N = 241), by presence of *International Classification of Diseases, Tenth Revision, Clinical Modification* (ICD-CM-10) discharge diagnosis code P96.1: Neonatal withdrawal symptoms from maternal use of drugs of addiction — selected hospitals, Pennsylvania, 2018

Characteristic	No.* (%) or mean (range)		p-value^§^
	Infants with NAS assigned discharge diagnosis code P96.1 (N = 174)^†^	Infants with NAS not assigned discharge diagnosis code P96.1 (N = 67)	
**Maternal race**			
White	153 (92)	58 (88)	0.379
Other^¶^	14 (8)	8 (12)	
**Maternal ethnicity**			
Hispanic or Latina	1 (1)	1 (2)	0.489
Not Hispanic or Latina	160 (99)	63 (98)	
**Source of payment in maternal record**			
Medicaid	158 (95)	58 (88)	0.048
Private/Other	8 (5)	8 (12)	
**Maternal age, yrs**	169**; 29 (18–41)	65**; 29 (19–43)	0.711
**Infant sex**			
Male	84 (48)	34 (51)	0.731
Female	90 (52)	33 (49)	
**Gestational age, wks**	169**; 38 (33–42)	66**; 38 (32–41)	0.683
**Type of hospitalization**			
Birth hospitalization	156 (90)	65 (97)	0.109
Other type of admission	17 (10)	2 (3)	
**Length of stay, days**	173**; 14 (1–68)	67**; 9 (2–47)	<0.001
**NAS scores**			
Age at first NAS score, days	168**; 2 (0–19)	66**; 1 (0–6)	0.031
First NAS Score^††^	173**; 4 (0–19)	66**; 2 (0–8)	<0.001
Age at highest NAS score, days	166**; 5 (0–32)	64**; 3 (0–10)	<0.001
Highest NAS score^††^	173**; 11 (2–21)	65**; 9 (2–16)	<0.001
**Symptoms**			
Total number of symptoms^§§^	173**; 12 (1–17)	67**; 9 (1–16)	<0.001
**Evidence of prenatal opioid exposure in the infant record^¶¶^**			
Neonatal toxicology evidence	114 (66)	43 (64)	0.845
Maternal toxicology evidence	90 (52)	30 (45)	0.334
Maternal history	161 (93)	64 (96)	0.567
**Evidence of prenatal opioid exposure in the maternal prenatal or delivery record^¶¶^**			
Maternal toxicology evidence	40 (23)	16 (24)	0.883
Maternal history	168 (97)	65 (97)	1.000
Type of opioid exposure***			
Buprenorphine	116 (67)	44 (66)	0.884
Methadone	53 (30)	15 (22)	0.212
Opiates, unspecified	50 (29)	19 (28)	0.954
Heroin	30 (17)	10 (15)	0.665
Oxycodone	21 (12)	9 (13)	0.774
Other opioids^†††^	11 (6)	6 (9)	0.474
**Type of other exposure** ^§§§^			
Tobacco	128 (74)	51 (76)	0.684
Cannabis	51 (29)	12 (18)	0.071
Cocaine	28 (16)	10 (15)	0.824
Antidepressants	30 (17)	5 (7)	0.054
Benzodiazepines	27 (16)	7 (10)	0.311
Amphetamine	23 (13)	4 (6)	0.169
Gabapentin	21 (12)	1 (1)	0.011
**Infant receipt of pharmacologic treatment for NAS**			
Yes	105 (61)	2 (3)	<0.001
No	66 (39)	63 (97)	
ICD-10-CM discharge diagnosis code****			
P04.1, Newborn (suspected to be) affected by other maternal medication	7 (4)	1 (1)	0.449
P04.2, Newborn affected by maternal use of tobacco	5 (3)	5 (7)	0.110
P04.3, Newborn affected by maternal use of alcohol	0 (—)	1 (1)	0.278
P04.41, Newborn affected by maternal use of cocaine	3 (2)	2 (3)	0.620
P04.49, Newborn (suspected to be) affected by maternal use of other drugs of addiction	40 (23)	40 (60)	<0.001

* Frequencies might not sum to total because of missing values.

^†^ Includes 135 cases identified through surveillance and 39 cases identified through diagnosis code only who were assigned discharge diagnosis code P96.1.

^§^ P-values were calculated comparing infants assigned P96.1 with infants not assigned P96.1 using a negative binomial likelihood ratio test for continuous variables and chi squared test (or Fisher’s exact test, if at least one cell count was <5) used for categorical variables.

^¶^ Other races included were Black, Asian, Native Hawaiian/Other Pacific Islander, American Indian/Alaska Native, not specified, or unknown. Given the small denominator in each category, all were collapsed into a single category.

** Data are not available for all members of this cohort.

^†† ^Includes all infants with a recorded score. All scores were Finnegan or modified Finnegan.

^§§ ^Signs and symptoms include tremors, breathing problems, blotchy skin, diarrhea, crying, fever, fussiness, gagging or retching, hiccups, hyperactive or exaggerated Moro reflex, frequent yawning, overactive reflexes, poor feeding, salivation, seizures, skin abrasions or excoriation, slow weight gain, sneezing, stuffy nose, suckling issues, sweating, vomiting, increased muscle tone, trouble sleeping, and any other symptom attributed to NAS by a clinician.

^¶¶ ^Not mutually exclusive categories.

*** As documented in the maternal record, infant record, or both.

^††† ^Other opioids include codeine, fentanyl, hydrocodone, hydromorphone, kratom, morphine, and tramadol.

^§§§ ^As documented in the maternal record, infant record, or both. Other substances with <20 infants exposed included alcohol, antipsychotics, barbiturates, bupropion, methamphetamine, phencyclidine, and other substances referred to directly, such as “methaqualone” or indirectly, such as “maternal polysubstance abuse.”

**** No infants were assigned ICD-10-CM code P96.2, “Withdrawal symptoms from therapeutic use of drugs in newborn,” or P04.40, “Newborn affected by maternal anesthesia and analgesia in pregnancy, labor and delivery.”

## Discussion

Based on medical record review at five hospitals, Pennsylvania’s NAS surveillance system had a PPV of 99% and sensitivity of 79%. Accurate NAS surveillance is necessary to monitor temporal and geographic changes in NAS incidence and to plan for needed services. Findings from this evaluation might inform NAS surveillance efforts in other states. First, ICD-10-CM code P96.1 was assigned to 71% of infants reported to Pennsylvania’s NAS surveillance system, demonstrating the utility of using this code to efficiently identify NAS cases. However, 78% of infants not reported to the system were also assigned P96.1. Infants who are assigned P96.1 meet the Council of State and Territorial Epidemiologists (CSTE) 2019 Tier 2 confirmed NAS case definition (*1*), which was released after this investigation. CSTE’s standardized definition might help clarify which infants should be reported for future surveillance efforts. Previous studies have found that use of P96.1 to identify infants with NAS can yield high PPV (*4*,*7*,*8*), and a combination of P96.1 or P04.49 improves sensitivity but decreases PPV (*5*). Second, in this investigation, infants with more severe signs and symptoms of NAS were more likely to be assigned P96.1. A recent review of surveillance practices highlighted the variability of NAS case definitions and use of ICD-10-CM codes across jurisdictions (*9*). Consistency in coding of infants with NAS could assist future surveillance efforts. Third, infants with toxicology evidence of prenatal opioid exposure were more likely to be reported to surveillance, but toxicology evidence was also frequently found among unreported cases. CSTE’s Tier 1 NAS confirmed case definition requires, in part, that infants have neonatal laboratory evidence of exposure (*1*); therefore, information on all infants with toxicologic evidence of exposure might warrant review when conducting NAS surveillance.

Although using P96.1 to trigger case review could have improved reporting to surveillance because it would have identified 78% of unreported NAS cases, using P96.1 as the sole criterion for reporting would have missed 29% of all infants reported with NAS. Medical record review was needed to identify infants with NAS who were less likely to have toxicology evidence of exposure (among infants not reported) and more likely to have less severe signs and symptoms of NAS (among infants not assigned P96.1). Therefore, these data suggest that using both diagnosis code assessment and focused medical record review as case-finding methods, though the latter might be labor intensive, would most accurately identify infants with NAS. Notably, in this investigation, this strategy relied on reviewing medical records of a selected group of infants with diagnosis codes indicative of prenatal substance exposure or a NAS symptom, and not only NAS diagnosis codes. Additional work is needed to identify the optimal subset of codes to identify possible infants with NAS (*5*,*7*,*8*).

CSTE released the first nationally standardized NAS case definition (*1*) after this investigation was completed; therefore, it could not be applied to these data. Differences include that the Pennsylvania NAS case definition included prenatal opioid exposure at any time during pregnancy, and the CSTE NAS definition includes not only exposure to opioids, but also benzodiazepines and barbiturates, and limits the exposure period to ≤4 weeks before delivery (*1*). Standardization of NAS reporting might improve with implementation of the CSTE definition.

The findings in this report are subject to at least three limitations. First, hospitals were selected to represent specific characteristics; these findings might not be representative of all hospitals in Pennsylvania or the United States. Second, in this investigation, NAS case status was determined based on infant charts alone, with maternal charts reviewed only among infants with confirmed NAS; findings might differ in investigations that can rely on both maternal and infant records to determine NAS case status. Finally, the estimate of the surveillance system’s sensitivity might be biased because this investigation focused on infants who possibly had NAS and did not include chart review for a sample of all infants; this would be needed to estimate true sensitivity.

Throughout the United States, NAS surveillance is in a nascent stage; NAS surveillance can be strengthened by using a combination of diagnosis code assessment and focused medical record review. Further evaluation of NAS surveillance systems after implementation of the CSTE case definition will be useful. Accurate NAS surveillance is needed to identify changes in incidence and regional differences and to plan for needed services.

**Summary**

**What is already known about this topic?**

Neonatal abstinence syndrome (NAS) has increased as part of the U.S. opioid crisis, but no national NAS surveillance system exists, and data about the accuracy of state-based surveillance are limited.

**What is added by this report?**

Among infants with confirmed NAS at five Pennsylvania hospitals, ICD-10-CM code P96.1 was assigned to 71% of those who were reported to the NAS surveillance system and 78% of those who were not reported to surveillance.

**What are the implications for public health practice?**

Accurate NAS surveillance, which is necessary to monitor changes and regional differences in incidence and assist with planning for needed services, includes a combination of diagnosis code assessment and focused medical record review.

## Footnotes

* As described in the Neonatal Abstinence Syndrome: 2018 Report released by the Pennsylvania Bureau of Epidemiology, the Pennsylvania NAS surveillance system was established through an emergency declaration that made NAS a reportable condition throughout the state. The Pennsylvania NAS surveillance case definition required health care providers at birthing facilities and pediatric hospitals to report infants born on or after January 10, 2018, to residents of Pennsylvania who received a diagnosis of NAS (based on prenatal exposure to opiate drugs anytime during pregnancy and the presence of at least one symptom of withdrawal) during the neonatal period (birth through 28 days of life). Reports were submitted through a web-based system.

† Included the following ICD-10 codes available as of October, 2018: F11.x; T40.0x–T40.4x, T40.6x, T50.7x; P96.1; P04.1x, P04.49, P04.89, P04.9; P04.2, P04.3, P04.41, P04.42, Q86.0; P90, R56.xx; P81.8, P81.9; R25.1, R25.8, R25.9; P94.1, P94.8, P94.9.

§ Signs and symptoms include tremors, breathing problems, blotchy skin, diarrhea, crying, fever, fussiness, gagging or retching, hiccups, hyperactive or exaggerated Moro reflex, frequent yawning, overactive reflexes, poor feeding, salivation, seizures, skin abrasions or excoriation, slow weight gain, sneezing, stuffy nose, suckling issues, sweating, vomiting, increased muscle tone, trouble sleeping, and any other symptom attributed to NAS by a clinician.

¶ 45 C.F.R. part 46.102(l)(2), 21 C.F.R. part 56; 42 U.S.C. Sect. 241(d); 5 U.S.C. Sect. 552a; 44 U.S.C. Sect. 3501 et seq.
